# Ethical Behavior in Organizations: Personal Values and the Moderating Role of Ethical Climate in Counterproductive Work Behavior and Organizational Citizenship Behavior

**DOI:** 10.3390/bs16030389

**Published:** 2026-03-08

**Authors:** Sergio Salgado, Carlos-María Alcover, Carolina González-Suhr

**Affiliations:** 1 Departamento de Administración y Economía, Universidad de La Frontera, Temuco 4811230, Chile; 2 Departamento de Psicología, Universidad Rey Juan Carlos, 28922 Madrid, Spain; carlosmaria.alcover@urjc.es

**Keywords:** ethical behavior, personal values, ethical climate, person-organization fit, moderating effect

## Abstract

This study analyzes the relationship between personal values and (un)ethical behavior in organizations, and the moderating role of perceived ethical climate. We integrate Schwartz’s theory of personal values with the Victor and Cullen model of ethical climate, following the recent reformulation proposed by Weber and Opoku-Dakwa, thereby offering a novel perspective not previously explored in empirical research. Relying on the Person–Organization Fit model, we test whether perceived ethical climate (specifically Egoism and Principled dimensions) moderates the relationship between personal values (Self-Transcendence and Self-Enhancement) and (un)ethical behavior, operationalized by Counterproductive Work Behavior (CWB) and Organizational Citizenship Behavior (OCB). To this end, we conducted a semi-longitudinal study involving a heterogeneous sample of workers from different organizations (Wave 1: *N* = 212; Wave 2: *N* = 84). The analyses supported that personal values and ethical climate are associated with (un)ethical behavior. Furthermore, significant interaction effects between ethical climate and personal values predicting CWB and OCB were found. This study contributes to a better understanding and management of ethical behavior, providing a theoretical contribution and plausible practical guidelines from a person-in-context approach. Limitations and challenges of this work are discussed.

## 1. Introduction

In times of uncertainty and economic turbulence, studying the factors that positively and negatively influence the ethical behavior of organizations is a particularly relevant concern (Potocan & Nedelko, 2021). As evidenced by the literature (e.g., Mishra et al., 2022; Moore & Gino, 2015; Paterson & Huang, 2019), public knowledge of numerous organizational malpractices (fraud, corruption, embezzlement, lies, or non-compliance with rules and laws) shows that promoting ethical behavior is a complex challenge. These difficulties remain despite the resources that companies invest in promoting it and the costs in terms of damaged corporate image, impaired prestige, and economic losses that these behaviors and practices entail, as well as increased absenteeism, lower job satisfaction, and high turnover due to employee distress (Cialdini et al., 2004). Consequently, the need to investigate the antecedents of ethical and unethical behavior continues to be a priority for academics and practitioners.

Research on behavioral ethics typically distinguishes between three streams: unethical behavior, compliance with minimum moral standards, and proactive ethical behavior (Treviño et al., 2006). In this study, we focus on the first and third streams, operationalizing (un)ethical behavior through two widely established constructs. Specifically, we examine Counterproductive Work Behavior (CWB) as a manifestation of unethical conduct—defined as intentional behavior that harms the organization or its members (Spector & Fox, 2005)—and Organizational Citizenship Behavior (OCB) as a form of ethical, prosocial behavior that supports the social and psychological environment of work (Organ, 1988). Adopting a deontological perspective (Jones, 1991), we interpret these constructs as actions inherently considered ‘correct’ or ‘incorrect’ within the organizational context. While CWB and OCB are distinct and not simply opposite ends of a continuum (Dalal, 2005), analyzing them jointly provides a comprehensive view of how personal values and climate promote ‘doing good’ (OCB) or inhibit ‘doing bad’ (CWB).

There is a consolidated body of previous evidence on individual variables and organizational factors that act as antecedents of (un)ethical work behaviors (e.g., Ambrose et al., 2008; Andreoli & Lefkowitz, 2009; Arnaud & Schminke, 2012; Baker et al., 2006; Treviño et al., 2014). Personal values are one of the individual factors analyzed as antecedents of both types of work behaviors (Feldman et al., 2015; Suar & Khuntia, 2010; Cai et al., 2024). In particular, Schwartz’s theory of personal values (Schwartz, 1992, 1994), defined as long-term goals that reflect the relevant aspects of people’s lives, can be a helpful theoretical framework for studying ethical and unethical behavior in organizations (Feldman et al., 2015). Personal values are central in shaping the sense of self, particularly in developing personal identity and the moral self, which is closely linked to the moral actor and ethical behaviors in social situations (Hitlin, 2011; Hitlin & Piliavin, 2004). However, despite their promising explanatory potential, personal values have hardly been used in research on ethical and unethical behaviors in organizations. Moreover, the results obtained have been inconsistent, so it is necessary to include other variables to clarify the differences previously found in research.

On the other hand, from the formulation of the ethical climate construct (Victor & Cullen, 1987, 1988), this theoretical framework is one of the most influential conceptual foundations in the field of business ethics in recent decades (Martin & Cullen, 2006; Simha & Cullen, 2012). Ethical work climate is a type of work climate that alludes to a set of climates of a prescriptive nature that capture organizational procedures, policies, and practices that have moral consequences for its members (Martin & Cullen, 2006). More specifically, “ethical climate is the perception of what constitutes right behavior, and thus becomes a psychological mechanism through which ethical issues are managed” (Martin & Cullen, 2006, p. 177). Although research on ethical climate and its measurement has widely used this theoretical framework (Newman et al., 2017), it is recognized that, both conceptually and operationally, this model of ethical climate has shown high variability in its psychometric performance. Based on the review and analysis of these issues, a reformulation of the model proposing a normative foundation for ethical work climate theory based on ethical philosophy and moral reasoning theory has recently been presented (Weber & Opoku-Dakwa, 2022). This framework seeks to incorporate a normative foundation that the original Victor and Cullen model did not contemplate. In this paper, we assume these recommendations in the partial operationalization of the model for this study.

Although prior literature has separately addressed the role of personal values and ethical climate in predicting (un)ethical behavior (e.g., Suar & Khuntia, 2010; Arciniega et al., 2019), their joint effects have not been empirically tested to date. Our study addresses this gap by integrating Schwartz’s theory of personal values with the recently revised Ethical Climate 2.0 model (Weber & Opoku-Dakwa, 2022). This novel combination allows us to test the moderating role of ethical climate in the relationship between values and ethical conduct from a person-organization fit perspective.

Regarding the scope of our model, we deliberately focused on specific dimensions of Schwartz’s values and ethical climate types rather than a comprehensive sweep. We selected Self-Transcendence vs. Self-Enhancement values and Principled vs. Egoism climates because they represent the theoretical antipodes of moral motivation (Schwartz, 2010; Victor & Cullen, 1988). Focusing on these ‘polar opposites’ allows us to test the mechanisms of congruence and incongruence in their most salient forms, providing a clearer test of the Person–Organization Fit dynamics than would be possible with more ambiguous or intermediate value dimensions.

Beyond identifying this empirical gap, the underlying research problem is conceptual in nature. While prior studies have examined values and ethical climate independently, the literature lacks a theoretically specified explanation of how ethical climates regulate the behavioral expression of personal values. Without such a framework, it remains unclear why similar value orientations may translate into divergent behavioral outcomes depending on organizational context. Accordingly, the guiding research question of this study is, How do contrasting ethical climate types regulate the translation of personal values into counterproductive and citizenship behaviors? Addressing this question enables us to move beyond additive models and to specify the regulatory mechanisms through which climate hierarchy shapes value-driven behavior within organizations.

## 2. Theoretical Background and Hypotheses Development

### 2.1. Personal Values and Unethical and Ethical Behaviors in Organizations

Values can have a cultural/societal and an individual character. The literature has debated how values influence people’s perceptions, cognitions, emotions, and behaviors (Beugelsdijk & Welzel, 2018; Sagiv & Schwartz, 2022; Schwartz, 2011). Regarding the influence of values on ethical and unethical behavior, a study conducted with participants from 48 countries (Ralston et al., 2014) concluded that values at an individual level had a greater weight in the explained variance of ethical behavior at work than values at a cultural/societal level. More specifically, the study conducted by Ralston et al. (2011), with data from 50 culturally and socioeconomically diverse societies, provided empirical evidence that Schwartz’s general values model is valid for analyzing ethical and unethical behaviors in work contexts.

In Schwartz’s value model (Schwartz, 1992, 2012), the two quadrants defining the individual’s orientation toward self-interest or interest in others are self-enhancement and self-transcendence. The values included in the self-enhancement quadrant are power and achievement, while universalism and benevolence comprise the self-transcendence quadrant. In addition, Schwartz’s model considers that these adjacent values share a motivational emphasis; thus, in the case of power and achievement, the underlying motivational drivers are social superiority and esteem, while in the values of universalism and benevolence, underlying motivations relate to the enhancement of others and the transcendence of selfish interests (Schwartz, 2012).

The evidence proves the opposite relationship between self-enhancement and self-transcendence values, especially in unethical behaviors, such as approval of cheating to satisfy the need for social approval motivated by adherence to power and achievement values (Pulfrey & Butera, 2013). This relationship between the normative opposites of individual value quadrants has been found in different contexts and unethical behaviors, such as academic fraud (Pulfrey & Butera, 2013) or doping in sports (Ring et al., 2022). Previous research shows that individuals with a high orientation toward power and self-enhancement values may be less receptive to contextual norms and codes of conduct and more likely to act in a risk-seeking fashion (C. Anderson & Galinsky, 2006). Thus, employees with high scores in self-enhancement values were more involved in unethical decision-making behaviors, that is, behaviors inconsistent with the ethical code of their profession, as well as in counterproductive work behaviors, such as cyberloafing (Arciniega et al., 2019). The evidence provided by the three extensive studies conducted by Feldman et al. (2015), with different samples and more than 40 societies represented, showed robust results about the relationship between individual values and attitudes and unethical behaviors. In particular, in all the studies, self-enhancement values, especially power, tended to be the most potent motivators behind unethical behaviors. However, the results regarding self-transcendence values were not as conclusive; whenever there was a significant effect, they were found to be in the expected direction. Thus, results show that the inhibitory effects of self-transcendence on unethical behavior were more pronounced when the harm was directed at others, i.e., when the victim of the unethical behavior was identifiable (Feldman et al., 2015). Moreover, as Sagiv and Schwartz (2022) emphasize, strikingly, despite cultural differences in ethical systems across societies, cultural or societal values did not moderate the associations between self-enhancement and self-transcendence individual values and unethical behaviors found by Feldman et al. (2015).

Research on workplace deviance and other forms of counterproductive work behavior (CWB) has been a central concern for decades due to the detrimental effects it can have on organizations, employees, and, indirectly, on societies as a whole (Mackey et al., 2021; Robinson & Bennett, 1995). CWB can be defined broadly as “behavior that harms organizations and people in organizations” (Spector, 2011, p. 342). More specifically, Gruys and Sackett (2003) proposed to define CWB as “any intentional behavior on the part of an organization member viewed by the organization as contrary to its legitimate interests” (p. 30). As with other types of unethical work behavior, attempts have been made to identify the antecedent factors of CWB, basically individual versus situational, and motivational versus control (e.g., Marcus & Schuler, 2004), or into four broad categories: personal, organizational, work, and contextual factors (e.g., Lau et al., 2003).

Although multiple individual antecedents have been examined, primarily personality traits, affect, and emotions (e.g., Fida et al., 2015; Spector et al., 2006; Whelpley & McDaniel, 2016), research on the relationships between individual values and CWBs is surprisingly scarce. Consequently, following the arguments and evidence presented above, this study attempts to identify the possible relationships between individual values and CWBs as unethical work behavior, for which we formulate the following hypotheses.

**H1a:**  *There will be a negative relationship between self-transcendence and unethical behavior in the form of CWB.*

**H1b:**  *There will be a positive relationship between self-enhancement and unethical behavior in the form of CWB.*

Regarding ethical behaviors at work, organizational citizenship behavior (OCB) has been the most analyzed by previous research during the last four decades. OCB refers to several positive behaviors at work, such as extra-role behaviors, prosocial behavior, or contextual performance (P. M. Podsakoff et al., 2000). Based on Organ’s (1988) initial definition, OCB is understood as voluntary, discretionary, individual behaviors that are not mandatory or contained in job requirements or contractual specifications, that are not formally recognized by organizational reward systems, and that are intended to benefit other members (coworkers, supervisors and so on) and the objectives of the organization as a whole (P. M. Podsakoff et al., 2000; Rotundo & Sackett, 2002). Meta-analytic results (e.g., N. P. Podsakoff et al., 2009) show that OCBs are positively related to several individual-level outcomes, such as managers’ ratings of employee performance and reward allocation decisions, and negatively associated with various quit-related criteria, such as employee turnover intentions, actual turnover, and absenteeism. At the organizational level, empirical evidence indicates a positive relationship with productivity, efficiency, cost reduction, and customer satisfaction and a negative relationship with turnover at the unit or team level. In addition, the positive relationship between OCB and performance at the workgroup level has also found meta-analytical support (Nielsen et al., 2009).

Typically, the literature establishes an opposition between OCB and CWB (Spector & Fox, 2002), assuming that both individual variables (e.g., personality traits, attribution style, attitudes, and other predispositions) and organizational conditions and constraints (e.g., job stressors, perceived organizational injustice, leader behaviors, violations of the psychological contract, or effort-reward imbalances) act as antecedents of positive emotions and reactions, which will increase the likelihood of OCB, or negative ones, which will increase the likelihood of CWB. Meta-analytic results (Dalal, 2005) indicate a modest negative correlation (*p* = −0.32) between OCB and CWB, and the strength of this relationship did not increase appreciably when the objective of both behaviors, i.e., the organization as a whole versus other employees, was the same (Dalal, 2005). In other words, and in conclusion, it is pertinent to analyze both constructs since they are not two extremes of the same variable but rather two types of behavior that must be managed differently based on their characteristics.

In analyzing the individual antecedents of OCB, only a limited body of research has been concerned with identifying relationships between personal values and these positive work behaviors, with research on personality traits, dispositional and attitudinal factors highly emphasized (Arthaud-Day et al., 2012; Tagliabue et al., 2020). However, while personality traits help explain which people tend to exhibit a specific behavior regularly, the advantage of personal values as an explanatory mechanism is that they allow us to understand the underlying factors behind behaviors, such as conscious thought or intentionality (Bilsky & Schwartz, 1994). Despite the promising potential of personal values to predict OCB, empirical analysis of this postulated relationship is very scarce, and in no case has it been systematically investigated (Arieli et al., 2020). Regarding self-transcendence values, previous research has found that benevolence predicts OCB toward the organization (Arthaud-Day et al., 2012). For their part, Cohen and Liu (2011) found that the value of benevolence predicted altruistic OCB, in the sense of being willing to perform helpful behaviors for coworkers, while Lönnqvist et al. (2006) found that the value of universalism was positively related to the altruistic behavior perceived by colleagues, one of the dimensions defining OCB. Indirect support for the relationships between self-transcendence values and ethical behavior at work can be found in the postulated relationship between this quadrant of values and ethical leadership (Banks et al., 2020). For instance, Illies and Reiter-Palmon (2008) found that individuals with self-enhancement values were more destructive in leadership roles than individuals with self-transcendence values; in addition, the core values of power (positive relationship with destructive behavior) and universalism (less positive relationship) were most influential.

As for the relationship between self-enhancement values and OCB, the research results are mixed. Although following the same argumentative logic as for CBW, an inverse relationship might be expected between them, some studies (Bolino et al., 2006; Rioux & Penner, 2001) have found a positive relationship based on the fact that employees motivated by impression management (a motive directly related to the power value) tend to show more OCB. However, other studies (Lönnqvist et al., 2006) found that power and achievement values were negatively related to altruistic behavior perceived by coworkers, while Arthaud-Day et al. (2012) found no significant relationship between power and OCB, which indirectly supports the expected negative relationship mentioned.

Based on these rationales, we propose the following hypotheses:

**H2a:**  *There will be a positive relationship between self-transcendence and ethical behavior in the form of OCB.*

**H2b:**  *There will be a negative relationship between self-enhancement and ethical behavior in the form of OCB.*

### 2.2. Moderation of the Ethical Climate in the Relationship Between Personal Values and (Un)Ethical Behavior

The study of ethical work climate has evolved significantly since Victor and Cullen’s (1988) seminal model. Initially, the construct comprised three ethical criteria—egoism (self-interest), benevolence (utilitarianism), and principle (laws and codes)—crossed with three loci of analysis (individual, organizational, and cosmopolitan) to operationalize how ethical decisions are determined (Martin & Cullen, 2006; Simha & Cullen, 2012).

However, recent scholarship argues for a more normative approach. Weber and Opoku-Dakwa (2022) proposed reforming the model to incorporate a hierarchical ethical preference, arguing that ethical behavior is inherently normative. Their Ethical Climate Questionnaire 2.0 (ECQ 2.0) offers a more parsimonious typology “by eliminating the individual locus of analysis and advocating for a normative hierarchical preference that can provide prescriptive guidance” (Weber & Opoku-Dakwa, 2022, p. 643).

This reformulation retains Egoism (pursuing personal gain and avoiding punishment) but refines the other dimensions. Benevolence is reconceptualized as a Conventional climate, focusing on in-group well-being, akin to Kohlberg’s conventional stage (Kohlberg, 1976), while Principled climate is redefined to focus on universal ethical principles such as rights, justice, and the greater good (post-conventional stage). In the present study, we adopt this updated framework. Specifically, we focus on the moderating effects of Egoism and Principled ethical climates. We selected these two because, conceptually and operationally, they represent the clearest antipodes of congruence and incongruence regarding Self-Enhancement and Self-Transcendence personal values, respectively.

Although the Ethical Climate 2.0 model (Weber & Opoku-Dakwa, 2022) includes intermediate dimensions such as Benevolence (associated with Conventional moral reasoning), the present study deliberately focuses on the Egoism and Principled dimensions. This choice is theoretically grounded in the authors’ normative reformulation, whereby these two climates correspond to the opposing poles of Kohlberg’s cognitive moral development framework: the Pre-conventional level (Egoism, oriented toward self-interest and instrumental reasoning) and the Post-conventional level (Principled, oriented toward universal ethical principles, justice, and rights). Focusing on these normative extremes allows for a more precise examination of Person–Organization Fit mechanisms. By contrasting the lowest and highest levels of organizational moral reasoning, the study isolates how a strong ethical situation (Principled climate) constrains self-interested tendencies, whereas a low-normative situation (Egoism climate) may fail to inhibit—or even amplify—unethical behavioral tendencies. This design directly responds to Weber and Opoku-Dakwa’s call to move beyond descriptive taxonomies toward a hierarchical, normatively grounded understanding of ethical climate and its behavioral consequences.

#### Integrating Personal Values and Climate: A Dynamic P-O Fit Approach

Previous research has established the moderating role of ethical climate on various individual outcomes (Newman et al., 2017), examining antecedents such as negative affect (Chen et al., 2013) or self-efficacy (Tanner et al., 2015). However, studies considering personal values as antecedents remain scarce. Although values and climate may share motivational content, they are profoundly distinct: values are stable, internal traits guiding individual behavior, whereas climate is a contextual interpretation of organizational norms (Schwartz, 2012; Victor & Cullen, 1987). This theoretical distinction supports the rationale for analyzing their interaction.

To understand their interaction, we ground our study in the Person-Organization (P-O) Fit framework (Chatman, 1989), which posits that behavior results from the congruence between individual attributes and organizational characteristics. However, we argue that “fit” is dynamic. A high fit with a negative environment may be pervasive, while “misfit” can trigger compensatory moral mechanisms. By integrating P-O Fit with the Value Activation Hypothesis (Verplanken & Holland, 2002) and Schwartz’s (1977) Normative Activation Model, we propose that the interplay between personal values and ethical climate creates distinct behavioral responses—Activation, Suppression, Neutralization, and Alignment—depending on whether the environment activates or deactivates the normative obligations associated with those values.

It is important to note that, although Schwartz’s model includes four higher-order value dimensions, this study focused on self-transcendence and self-enhancement because previous research has emphasized these dimensions as particularly predictive of moral action (Schwartz et al., 2012), representing the dimensions most directly relevant to predicting OCB and CWB. Furthermore, the person–organization fit perspective suggests that incongruent value–climate configurations may be especially relevant for understanding value–behavior dynamics, as they create psychological tension that triggers adjustment (Cable & Edwards, 2004).

Accordingly, we formulate the following hypotheses.

*Moderation by Value Activation (Self-Transcendence × Egoism → CWB).* From a P-O Fit perspective, a high Egoism climate represents a fundamental value incongruence for employees with high Self-Transcendence (ST). When an organization promotes egoism, it violates the self-concept of a high-ST individual. According to Value Activation Theory (Verplanken & Holland, 2002) and Trait Activation Theory (Tett & Guterman, 2000), this specific value threat does not lead to assimilation. Instead, it makes the central value cognitively salient. The mismatch triggers an activation of the central values: the employee asserts their self-concept against the environment to maintain internal consistency. Thus, while low-ST employees experience congruence with the egoism climate (facilitating CWB), high-ST employees experience central value activation, inhibiting deviant behavior more strongly than they would in a neutral setting. Aquino et al. (2009) showed that situational primes for self-interest failed to influence individuals whose moral identity was central to their self-concept. Similarly, Greenbaum et al. (2012) demonstrated that employees with strong moral traits can ‘uncouple’ themselves from unethical environments, as their internal standards of conduct override situational pressures. Furthermore, Skarlicki et al. (2008) found that individuals with high moral identity were less likely to retaliate even when provoked, acting as a buffer against situational triggers for CWB. Collectively, these findings reinforce the expectation that for high-ST individuals, an egoism climate triggers a value-activation response, inhibiting CWB through the assertion of their central self-concept against the environment.

**H3a:**  *Egoism ethical climate will be positively related to unethical behavior in terms of CWB.*

**H3b:**  *Egoism ethical climate moderates the relationship between Self-Transcendence and CWB. High ST individuals will exhibit a value activation response, showing significantly lower CWB under high egoism compared to low ST individuals.*

*Moderation by value suppression (Self-Transcendence × Egoism → OCB).* While P-O Fit theory posits that congruence fosters extra-role behaviors like OCB, the nature of the climate matters. Even if an individual possesses the values for OCB (High ST), an Egoism climate disrupts the normative activation process. Combining Schwartz’s (1977) concept of Awareness of Consequences with motivation crowding theory (Frey, 1997), we argue that an egoism climate imposes a “transactional frame” that suppresses intrinsic motivations. The environment signals that altruism is naïve or instrumental, creating a Suppression effect. This reduces the awareness of others’ needs, blocking the expression of ST values. Thus, despite the individual’s potential for fit in terms of capability, the climate severs the link between prosocial values and citizenship behavior. In this vein, for example, Vohs et al. (2006) demonstrated that priming money (egoism) reduced helping behavior, and Tenbrunsel and Messick (1999) found that framing situations as “business decisions” suppressed cooperative tendencies.

**H4a:**  *Egoism ethical climate will be negatively related to ethical behavior in the form of OCB.*

**H4b:**  *Egoism ethical climate moderates the relationship between Self-Transcendence and OCB through suppression. The positive relationship between ST and OCB will be significantly weaker (or non-significant) under high egoism climate conditions.*

*Moderation by value neutralization (Self-Enhancement × Principles → CWB). Individuals* with high Self-Enhancement (SE) typically exhibit low fit with ethical norms, often leading to CWB. However, P-O Fit literature acknowledges that strong situations can restrict the expression of personality (Cooper & Withey, 2009). A Principled Climate creates a structural constraint that forces behavioral compliance. By imposing strict rules and sanctions—thereby enforcing a high Ascription of Responsibility (Schwartz, 1977)—the climate neutralizes the natural tendency of high-SE employees to prioritize the achievement of personal well-being, which has been linked to the occurrence of misbehavior. Here, the organization overrides the “P” factor, resulting in neutralization. The climate buffers the effect of values, ensuring that even self-interested employees behave as if they fit the ethical standards. Accordingly, for example, Kish-Gephart et al. (2010) indicated that code enforcement weakens the link between self-serving individuals and unethical choices, and Winter et al. (2004) found that explicit rule structures reduce the predictive power of self-interested motives.

**H5a:**  *Principled ethical climate will be negatively related to unethical behavior in terms of CWB.*

**H5b:**  *Principled ethical climate moderates the relationship between Self-Enhancement and CWB. The positive relationship between SE and CWB will be neutralized (non-significant) under high principled climate conditions.*

*Moderation by value alignment (Self-Enhancement × Principles → OCB). Finally*, we explore whether a Principled Climate can induce prosocial behavior in self-interested individuals. High SE individuals are driven by external rewards and status. In a climate where adherence to principles is the dominant metric of success, engaging in OCB may become a strategy for impression management (Bolino, 1999). We propose that the Principled Climate aligns the self-interest of the individual (SE) with the functional goals of the organization (Kristof-Brown et al., 2005). Through this functional alignment, the climate might transform the egoism drive into outward citizenship behavior, not out of altruism, but as a calculated effort to achieve fit and avoid social sanctions. In line with this idea, Grant and Mayer (2009) identified the “Good Soldier Syndrome,” where self-interested motives drive prosocial behavior if perceived as instrumental, and Rioux and Penner (2001) found that OCB is often driven by impression management in regulated environments.

**H6a:**  *Principled ethical climate will be positively related to ethical behavior in the form of OCB.*

**H6b:**  *Principled ethical climate moderates the relationship between Self-Enhancement and OCB. Specifically, the relationship between SE and OCB will become more positive (or less negative) under high principled climate conditions compared to low principled climate conditions.*

**Figure 1:**
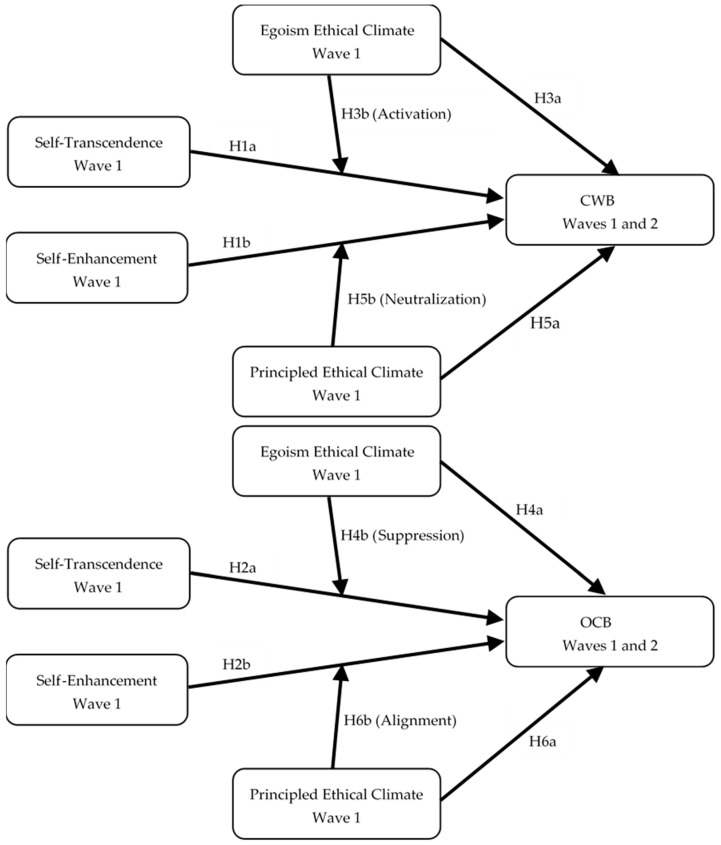
Hypothetical conceptual model. The diagram illustrates the direct effects of ST and SE on CW and OCB, and the moderating effects of egoism and principled climate on these relationships. Direct effects of ethical climates on CWB and OCB are represented too.

Figure 1 depicts the conceptual model underlying this study. The model proposes direct effects of self-transcendence (ST) and self-enhancement (SE) on counterproductive work behavior (CWB) and organizational citizenship behavior (OCB), as well as moderating effects of egoism and principled ethical climates on these relationships.

## 3. Materials and Methods

### 3.1. Procedure and Participants

According to the purpose of the study, a semi-longitudinal correlational study was conducted in two waves on a non-probabilistic sample aimed at a general working population. In total, 212 and 84 participants completed the study in the first and second waves, respectively.

The sample size for Wave 1 (*N* = 212) was deemed sufficient for the proposed analyses, exceeding the recommended minimum ratio of 10 participants per estimated parameter for regression-based models (Hair et al., 2014) and providing adequate statistical power (0.80) to detect medium effect sizes (*f*^2^ = 0.15) at *α* = 0.05.

In the first wave, all the variables under study were included, while in the second wave, only the criterion variables were included. This second wave was carried out between three and five months after the first participation in the group of participants who agreed to receive a follow-up on the study. The demographic information of the participants is presented in Table 1. As can be seen, the sample is balanced in terms of gender and other demographic characteristics. All participants lived in Chile at the time of the study. The data for the first wave was collected between September and December 2021, while the second wave was carried out between January and May 2022. To link the data collected in Waves 1 and 2, each participant provided a password consisting of the first five digits of the national identity card (ID) number plus the initial of the first name and the initial of the last name, reinforcing the privacy and confidentiality of the information.

Regarding the first wave, it was important to control the common method bias (CMB) effect using a priori and post hoc strategies. Regarding the a priori strategies, instruments with different scale properties were used, which helps to prevent CMB (P. M. Podsakoff et al., 2012, 2024). Specifically, the personal values questionnaire asks respondents to evaluate how similar certain personal profiles—which denote values, principles, or interests—are to their own. In contrast, the ethical climate questionnaire asks to what extent it is true or false that certain ethical criteria are considered in the organization’s decision-making. Meanwhile, the OCB measure asks respondents to evaluate the extent to which certain behaviors are characteristic of their own conduct, and the CWB measure, on the other hand, requests information on the frequency with which they perform certain negative actions. In this way, each questionnaire places the person in a different evaluation mode, preventing routinization, acquiescence, and ultimately, the risk of CMB (the questionnaires are presented in Appendix A, Table A1, Table A2 and Table A3). Moreover, the study design separates the independent variables (personal values and ethical climate) psychologically from the criterion variables (ethical and unethical behavior) through a distraction task (sort a list of five well-known multinational companies from the one with the most to the one with the fewest direct and indirect workers in the world). Including a distraction task decreases the probability of finding correlations between independent and criterion variables due to the variance of the common method or the search for consistency between responses (see Section 3.2 for post hoc analyses). Additionally, measuring only the criterion variables in the second wave complements these a priori strategies, avoiding the risk of CMB and adding predictive and discriminant validity to the study results.

The recruitment was conducted online using the Questionpro platform, following a snowball strategy that started with a combination of professional networks, social networks, professional associations, undergraduate and graduate students, and personal contacts, who were invited to participate and disseminate this invitation among their network of relations. Participants took an average of 25.8 and 6.7 min to complete the study in Waves 1 and 2, respectively.

The project underwent assessment by the Ethics and Bioethics Committee of the Universidad de La Frontera (N° 015/20, 24 June 2020). Consequently, all participants were asked to read an informed consent before starting the study. When the proposed conditions were agreed upon, the participants marked a square indicating they were over 18 years old, had been informed, understood the nature of this study, and had decided to participate voluntarily.

### 3.2. Data Analysis

First, we examined univariate and multivariate normality. For univariate normality, cut-off values of ±3 for skewness and ±7 for kurtosis were considered acceptable (Curran et al., 1996; DeCarlo, 1997). Multivariate normality was also evaluated, since departures from normality may affect structural modeling.

Second, we conducted confirmatory factor analyses (CFA) using the unweighted least squares method (ULS; Muthén, 1993) with robust error estimation. This method is recommended for small samples and is robust to deviations from normality (Rhemtulla et al., 2012). Model fit was evaluated using the following criteria: *χ*^2^/*df* ratio < 3, RMSEA ≤ 0.06, SRMR ≤ 0.08, CFI ≥ 0.95, and TLI ≥ 0.95 (Hu & Bentler, 1999). Internal consistency reliability of the scales was tested using Cronbach’s alpha (α) and McDonald’s omega (ω). While values exceeding 0.70 are generally preferred in the social sciences (Hair et al., 2014; Lance et al., 2006), values above 0.60 were deemed acceptable for this study. This lower threshold is consistent with methodological recommendations for short scales with broad conceptual bandwidth (Loewenthal, 2001) and is in line with extensive research using Schwartz’s value scales, where reliabilities in the 0.60–0.70 range are commonly reported and accepted due to the abstract nature of the constructs (Davidov et al., 2008; Schwartz et al., 2001; Schwartz, 2021).

Third, we analyzed discriminant validity. Following the Fornell–Larcker criterion, the correlation between constructs should be lower than the square root of the AVE of each latent construct. Additionally, confirmatory factor analyses (CFA) were conducted in two steps: (a) testing the fit of a single-factor model in which all items load on a common latent factor, and (b) testing the fit of the theoretical multi-factor model. Discriminant validity is supported when the single-factor model shows poor fit while the theoretical model demonstrates acceptable fit (J. C. Anderson & Gerbing, 1988).

Fourth, we assessed the potential impact of common method bias (CMB). As a post hoc procedure, we conducted Harman’s single-factor test through exploratory factor analysis. This widely used approach evaluates whether a single unrotated factor explains more than 50% of the total variance; if so, CMB may represent a concern (P. M. Podsakoff et al., 2024; Tehseen et al., 2017). Additionally, we examined CMB by inspecting the correlation pattern among the theoretical constructs. Following the recommendations for ULS estimation (Tehseen et al., 2017), we reviewed the inter-construct correlations for the six variables analyzed.

Fifth, to ensure that collinearity did not bias the moderation models, we planned to calculate Variance Inflation Factors (VIFs) for all predictors and interaction terms. Following Hair et al. (2014), VIF values below 5 were considered acceptable, ruling out detrimental multicollinearity.

Sixth, an attrition test was conducted to verify that no systematic bias existed between the group that participated at Wave 1 and the group that completed Wave 2 in this semi-longitudinal study.

Lastly, linear regression analyses were performed to test the hypotheses. For processing data, JASP version 0.18.3 and Lavaan version 0.4-14 were used (JASP Team, 2024; Rosseel, 2012).

### 3.3. Instruments

Personal values were measured using the Portrait Values Questionnaire (PVQ; Schwartz et al., 2001) validated in Spanish by Beramendi and Zubieta (2017). This questionnaire includes 21 items (see Appendix A, Table A1). Self-transcendence (ST) is measured through five items (e.g., “She/he thinks it is important that every person in the world be treated equally. She/he believes everyone should have equal opportunities in life.”), and Self-enhancement (SE) is measured through four items (e.g., “It is important to her/him to be rich. She/he wants to have a lot of money and expensive things.”). Both are scored on a 6-point Likert scale (1 = Not like me at all, to 6 = Very much like me).

Ethical climate was measured by the Ethical Climate Questionnaire (Victor & Cullen, 1988; Cullen et al., 1993), adapted following the recommendations of Weber and Opoku-Dakwa (2022). This instrument includes 36 items. Six items measure egoism ethical climate (e.g., “In this organization, people protect their interests”). Six items measure the perception of principled ethical climate (e.g., “When making decisions, people in this organization are encouraged to weigh justice, fairness, human rights, and the effects of their actions on the greater good”). Both scales are presented in Appendix A (Table A2). The participants scored to what extent each statement was true in its organization on a 6-point Likert scale (1 = Mostly false, to 6 = Completely true).

Ethical conduct was operationalized through the OCB measure introduced by Kelloway et al. (2002), adapted to Spanish (see Appendix A, Table A3). This instrument contains nine items (e.g., “Volunteering to do things not formally required by the job”). The respondents used a five-point Likert scale (1 = not at all characteristic, and 5 = very characteristic) to indicate the extent to which each item was characteristic of oneself.

Unethical conduct was operationalized through the CWB measure introduced by Kelloway et al. (2002), adapted to Spanish (see Appendix A, Table A3). This instrument includes ten items. Respondents were asked to indicate how often they had engaged in each of the listed behaviors (e.g., “Gossiped about your co-workers,” “Exaggerated about your hours worked.”) on a five-point Likert scale of 1 (from 1 = never to 5 = very often).

## 4. Results

Table 1 presents the demographic information of the participants. While individuals with higher education are more represented, the sample is generally heterogeneous in terms of reported characteristics. It is noteworthy that, despite the sample loss at Wave 2, the proportions of participants in the total sample for each demographic category were maintained, indicating that there is no apparent bias that prevents reporting the results from both waves, which constitutes a significant contribution to the objectives of this research. It is also worth remembering that all variables were measured at Wave 1, but a second measurement of the dependent variables was only included at Wave 2 to avoid detrimental effects such as common method variance or response acquiescence.

To assess potential attrition bias, we conducted independent-samples *t*-tests comparing Wave 1 participants who completed Wave 2 (*n* = 84) with those who did not (*n* = 128) on the main study variables. No significant differences were found for self-enhancement, egoism climate, principled climate, OCB, or CWB (all *p* > 0.22). However, participants who dropped out showed slightly higher self-transcendence scores at Wave 1 (*p* = 0.018). The lack of systematic differences supports the suitability of using Wave 2 data to examine longitudinal effects.

*Univariate and multivariate normality.* The items of the scales revealed acceptable values of skewness and kurtosis, except for two items of self-transcendence that showed a kurtosis over 4 (“He/she thinks it is important that every person in the world be treated equally. He/she believes everyone should have equal opportunities in life”; “It is important to him/her to be loyal to his/her friends. He/she wants to devote herself to people close to him/her.”); and one item of the CWB (“Gossiped about your coworkers”). In both cases, responses were accumulated in scores representing positive behavior. On the other hand, all the scales showed acceptable skewness and kurtosis values, except for CWB, which showed a slightly high kurtosis (7.57). These results indicate that the assumption of univariate and multivariate normal distribution regarding CFA is unmet. However, the composite variables (scales) showed acceptable univariate and multivariate normal distribution values according to the linear regression analysis. Given the use of robust ULS estimation, these deviations do not compromise parameter estimation.

*Internal consistency reliability.* Results showed acceptable internal consistency levels when observing Cronbach’s alpha and McDonald’s omega. Scales showed alpha and omega values over 0.75, except for self-transcendence, which, in line with previous literature (Schwartz, 2021), presents values over 0.64 for both statistics. Cronbach’s alpha (α), MacDonald’s omega (ω), means, standard deviations and variable correlations are shown in Table 2.

*Common method bias (CMB) and discriminant validity*. Harman’s test showed that the first unrotated factor (eigenvalue > 1) explains 20.34% of the variance. In addition, the inter-correlations among the study variables were generally low to moderate (average *r* = 0.19, range = 0.007 to 0.392; see Table 2), further suggesting that common method bias is unlikely to have substantially influenced the results. Regarding discriminant validity, the diagonal of Table 2 presents the square root of AVE; the square root of the AVE was greater than the latent variable correlations in all cases, which supports discriminant validity. Additionally, to assess the potential impact of multicollinearity—particularly within the moderation models—variance inflation factors (VIFs) were calculated using mean-centered predictors and interaction terms. All VIF values ranged from 1.02 to 1.35, which is well below the recommended threshold of 5 (Hair et al., 2014).

*Confirmatory factor analysis (CFA)*. We conducted confirmatory factor analyses (CFA) using the unweighted least squares method (ULS; Muthén, 1993) with robust error estimation. This method is recommended for small samples and is robust to deviations from normality (Rhemtulla et al., 2012). As shown in Table 3, this analysis was performed in three stages. First, the fit of the one-factor model to the data was checked, and then the fit of the theoretical model was verified. The results support the theoretical structure of the model, providing discriminant validity. We discarded four items from the initial theoretical six-factor model—one for each moderator variable (see Appendix A, Table A2) and one for each criterion variable—that had lower factor loadings (<0.219; see Appendix A, Table A3). The final six-factor model showed an acceptable fit.

*Hypothesis testing*. Table 4 presents the results of the linear regression analyses testing the hypothesized direct and interaction effects for Waves 1 and 2. All predictors and outcome variables were standardized prior to analysis. VIFs are reported for each model, and R^2^ values are shown for models including interaction terms. All the models included age and gender as control variables (these results are reported only if significance is found).

Regarding Hypothesis 1a and 3a, results revealed a significant negative effect of self-transcendence (ST) on counterproductive work behavior (CWB) (Wave 1: *β* = −0.261, *p* < 0.001; Wave 2: *β* = −0.289, *p* = 0.007), and a positive effect of egoism ethical climate on CWB (Wave 1: *β* = 0.140, *p* = 0.038; Wave 2: *β* = 0.252, *p* = 0.016). These findings are consistent with prior research indicating that prosocial values reduce the likelihood of deviant behavior (e.g., Schwartz, 1992), while egoism climates can foster instrumental or self-serving behaviors (Victor & Cullen, 1988).

As expected in Hypothesis 3b, the interaction between ST and egoism climate significantly predicted CWB (Wave 1: *β* = −0.255, *p* < 0.001, *R*^2^ = 0.160; Wave 2: *β* = −0.513, *p* < 0.001, *R*^2^ = 0.411). This suggests that the negative relationship between ST and CWB is stronger in egoism climates, possibly because individuals with high ST experience dissonance in such settings, triggering moral action. These results support the idea that the misfit between personal values and organizational ethical context affects outcomes (Brown & Treviño, 2006).

For Hypothesis 2a, ST showed a positive effect on organizational citizenship behavior (OCB) in Wave 1 (*β* = 0.385, *p* < 0.001), but was marginally significant in Wave 2 (*β* = 0.211, *p* = 0.070). Egoism climate did not significantly predict OCB in either wave (H4a). Regarding Hypothesis 4b, a significant interaction was found between ST and egoism climate in both waves (Wave 1: *β* = −0.135, *p* = 0.050, *R*^2^ = 0.178; Wave 2: *β* = −0.259, *p* = 0.036, *R*^2^ = 0.111). The pattern suggests that ST leads to more OCB in low egoism climates, but its influence weakens under higher levels of egoism climate. This is consistent with research on moral licensing and climate suppression effects (Shao et al., 2008).

Turning to Hypotheses 1b and 5a, self-enhancement (SE) had a strong and positive effect on CWB (Wave 1: *β* = 0.318, *p* < 0.001; Wave 2: *β* = 0.325, *p* = 0.003), while principled climate had a negative effect (Wave 1: *β* = −0.151, *p* = 0.023; Wave 2: *β* = −0.214, *p* = 0.040). This supports the idea that individuals driven by achievement and power may engage in norm-breaking behavior, while ethical principles embedded in the organization serve as a protective factor.

Consistent with Hypothesis 5b, the interaction between SE and principled climate significantly predicted CWB in both waves (Wave 1: *β* = −0.133, *p* = 0.043, *R*^2^ = 0.136; Wave 2: *β* = −0.261, *p* = 0.016, *R*^2^ = 0.240). These findings suggest that principled climates weaken the influence of self-enhancing values on deviant behavior, consistent with the notion of ethical buffering or constraint mechanisms (Treviño et al., 2006).

Finally, Hypotheses 2b and 6b were not supported. The overall model explained 10.6% of the variance in OCB in Wave 1 and 4.7% in Wave 2. Consistent with H6a, Principled climate showed a significant positive effect on OCB in Wave 1 (*β* = 0.285, *p* < 0.001), although this effect did not replicate in Wave 2. Regarding H2b, although Self-Enhancement was hypothesized to negatively predict OCB, the observed coefficients were positive but non-significant in both waves. This unexpected direction is discussed further in light of prior research suggesting that self-enhancement may be associated with OCB under certain instrumental conditions (Bolino et al., 2006; Rioux & Penner, 2001).

**Figure 2:**
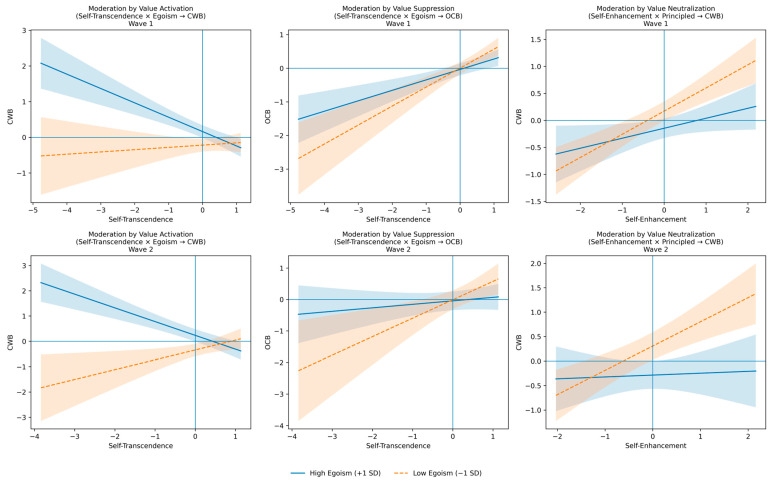
Moderation effects by activation, suppression and neutralization on Waves 1 and 2. *Note.* The plots depict simple slopes analyses for the significant interactions. Solid lines represent high levels of the moderator (+1 SD), while dashed lines represent low levels of the moderator (−1 SD). The shaded areas indicate 95% confidence intervals. Abbreviations: CWB = Counterproductive Work Behavior; OCB = Organizational Citizenship Behavior; SD = Standard Deviation.

To clarify the nature of the significant interaction effects reported in Table 4, we conducted a Simple Slope Analysis (Aiken et al., 1991) examining the relationship between personal values and behavior at high (+1 SD) and low (−1 SD) levels of the moderator. Table 5 and Figure 2 summarize and represent these results, contrasting the cross-sectional (Wave 1) and longitudinal (Wave 2) patterns.

*Moderation by Value Activation (Self-Transcendence × Egoism → CWB).* Regarding the interaction between Self-Transcendence (ST) and Egoism Climate on CWB, the analysis revealed a clear pattern of value centrality activation. In Wave 1, the relationship between ST and CWB was non-significant under a low Egoism climate (*β* = 0.07, n.s.), but became strongly negative and statistically significant under a high Egoism climate (*β* = −0.40, *p* < 0.001). This value-challenging situation, which activates the centrality of ST values, intensified over time: in the longitudinal analysis (Wave 2), the protective slope under high Egoism became even steeper (*β* = −0.51, *p* < 0.001), explaining a substantial portion of unique variance (Δ*R*^2^ = 0.20). Theoretically, this indicates that distinct configurations of Person-Environment fit drive behavior. In a high Egoism climate, employees with low Self-Transcendence experience congruence, reinforcing their tendencies and resulting in the highest observed levels of CWB. Conversely, under conditions of high Self-Transcendence, the high egoism climate creates a contrast effect that renders personal values more salient. In the absence of an ethically supportive context, high-ST employees are compelled to rely more heavily on their internal value system to guide behavior, whereas in low-egoism climates, this internal regulation is less critical. This supports the value activation hypothesis: the value predicts behavior most strongly when the context makes it central to decision-making.

*Moderation by value suppression (Self-Transcendence × Egoism → OCB).* The results for the interaction between ST and Egoism Climate on OCB provide compelling evidence of a value suppression, which became fully evident in the longitudinal data. In Wave 1, Self-Transcendence was positively related to OCB at both levels of the moderator, though the relationship was significantly stronger under a low Egoism climate (*β* = 0.56, *p* < 0.001) compared to a high Egoism climate (*β* = 0.31, *p* < 0.001). However, Wave 2 revealed the full extent of the suppression effect. While the relationship remained robust in the low Egoism climate (*β* = 0.57, *p* = 0.006), the high Egoism climate completely neutralized the link between values and prosocial behavior (*β* = 0.10, n.s.). This indicates that while high ST individuals are naturally inclined toward OCB, a persistent egoism environment acts as a structural constraint, effectively dampening the expression of positive values over time.

*Moderation by value neutralization (Self-Enhancement × Principles → CWB).* Finally, the interaction between Self-Enhancement (SE) and Principled Climate on CWB confirmed a neutralization (or buffering) hypothesis. The positive association between SE and CWB was strong and significant when the Principled Climate was low in both Wave 1 (*β* = 0.42, *p* < 0.001) and Wave 2 (*β* = 0.50, *p* < 0.001). Notably, this relationship was effectively neutralized when the Principled Climate was high. This buffering effect was marginal in the cross-sectional analysis (*β* = 0.18, *p* = 0.060) but became completely non-significant in the longitudinal analysis (*β* = 0.04, n.s.). This suggests that a strong principled environment effectively overrides the potential negative impulses associated with self-enhancement values, rendering them non-predictive of deviant behavior.

The observed increases in explained variance (Δ*R*^2^) by the interaction terms are consistent with the literature in social and organizational psychology, where interaction effects are typically difficult to detect due to measurement error and statistical power limitations (McClelland & Judd, 1993). Indeed, meta-analytic reviews indicate that the median effect size (*f*^2^) for interactions in social and behavioral sciences often falls between 0.002 and 0.009 (Aguinis et al., 2005; Evans, 1985). Therefore, our findings (ranging from Δ*R*^2^ = 0.016 to 0.20; see Table 5) are considered theoretically and practically significant.

## 5. Discussion

In this study, we addressed the dynamics of (un)ethical behavior in organizations by proposing a novel integration of Schwartz’s personal values theory and the perceived ethical climate model. Beyond a traditional congruence perspective, we adopted a Dynamic Person–Organization (P-O) Fit approach (Chatman, 1989) and enriched the Value Activation Hypothesis (Verplanken & Holland, 2002) and Schwartz’s (1977) Normative Activation Model. We posited that the relationship between personal values and behavior is not static but is moderated by the organizational context. Under this framework, the ethical climate acts as a situational trigger that either activates or deactivates the normative obligations associated with personal values. The longitudinal confirmation of the hypothesized mechanisms—Activation, Suppression, and Neutralization—offers strong support for this interactionist perspective.

Regarding the measurement of the organizational context, this work makes a specific theoretical and empirical contribution by validating the normative reformulation of the ethical climate model proposed by Weber and Opoku-Dakwa (2022). Unlike the original descriptive model (Victor & Cullen, 1988), this study operationalized the Egoism and Principled climates as hierarchical normative preferences (see Appendix A, Table A2). We focused on these two dimensions because, as argued in our theoretical framework, they represent the clearest antipodes of value–climate configuration: Egoism as the driver of self-interest (challenging Self-Transcendence) and Principled climate as the structural guardian of ethics (constraining Self-Enhancement). Our results support the psychometric validity of this updated approach, suggesting that measuring climate through a normative lens offers a more precise understanding of how organizational signals shape individual moral agency. While we focused on these core dimensions, future work should extend this validation to the other climate types contemplated in the model and adapt this instrument to specific contexts (e.g., Aldazabal et al., 2017).

Regarding the direct effects of personal values, our findings confirm the motivational conflict inherent in Schwartz’s model. Consistent with Hypotheses H1a and H2a, Self-Transcendence—characterized by concern for the welfare of others—emerged as a robust predictor of ethical functioning, significantly inhibiting Counterproductive Work Behavior (CWB) and promoting Organizational Citizenship Behavior (OCB). This supports the view that self-transcendent values provide an internal moral compass that operates across different contexts (Feldman et al., 2015). Conversely, and consistent with Hypothesis H1b, Self-Enhancement was a strong positive predictor of CWB, confirming that the pursuit of power and dominance, when unchecked, facilitates norm-breaking behaviors (Arciniega et al., 2019).

In terms of the organizational context, the results largely supported the theoretical premises of the reformulated Ethical Climate 2.0 model. As predicted in Hypothesis 3a, an Egoism climate was positively related to CWB, suggesting that environments emphasizing self-interest provide a justification for deviant behavior. In contrast, a Principled climate (Hypotheses 5a and 5b) functioned as a protective factor, negatively predicting CWB and positively predicting OCB (in Wave 1), validating the notion that clear ethical standards constrain misconduct and encourage compliance.

Regarding the non-significant direct relationship between Egoism climate and OCB (H4a), this may be due to the nature of OCB as a discretionary behavior. An Egoism climate creates pressure to prioritize self-interest, which strongly predicts CWB (as seen in H3a), but it does not necessarily preclude employees from performing occasional extra-role behaviors if they perceive them as instrumental for their own reputation or career advancement (Bolino, 1999). Thus, the suppression of OCB in an Egoism climate appears to be more dependent on the interaction with personal values (as shown in the moderation results) than on the direct main effect of the climate itself.

Regarding the interaction between Self-Transcendence (ST) and Egoism on CWB (H3b), the results reveal a mechanism of ethical activation. The simple slope analyses show that the protective effect of ST values was specifically activated by the high-Egoism climate. From a P-O Fit perspective, a high-Egoism climate represents a moral threat to self-transcendent individuals. This misfit appears to trigger a normative activation process: the environmental pressure to act selfishly makes the individual’s internal moral standards more salient. Consequently, high-ST employees actively engage in low deviance levels. In contrast, low-ST employees, lacking this internal anchor, drift toward higher CWB, effectively complying with the Egoism norms. Furthermore, these results align with the findings of Van den Broeck et al. (2011), who observed that an intrinsic work value orientation acts as a personal resource that strengthens employees’ resilience against demanding environments. From this perspective, Self-Transcendence functions not merely as a moral preference, but as a robust cognitive resource. This ‘moral resource’ effectively buffers the pressure of an Egoism climate, allowing high-ST individuals to maintain ethical consistency and inhibit CWB, even when situational cues encourage self-interest.

Conversely, regarding OCB (H4b), the results illustrate how an incongruent environment can suppress positive value expression. While self-transcendence is theoretically a consistent driver of prosocial behavior, our longitudinal data (Wave 2) showed that a high-Egoism climate effectively neutralized this relationship. This finding supports the value suppression hypothesis. OCB is discretionary and governed by prescriptive morality (doing good). In an Egoism climate, where self-interest is the norm, prosocial actions may be viewed as counter-normative, naive, or simply unrewarded. Thus, despite the individual’s intrinsic motivation (high ST), the organizational signals deactivate the behavioral expression of these values. The environment creates a situational constraint that prevents moral intent from translating into moral action.

The interaction between Self-Enhancement (SE) and Principled Climate on CWB (H5b) offers a distinct theoretical insight: the power of situational strength. Our results confirm that a Principled climate acts as a buffering mechanism against the “dark side” of self-enhancement. In low-principled contexts (weak situations), behavior is driven by individual traits, allowing the self-interested tendencies of SE to manifest as CWB. However, high-principled climates—characterized by clear rules, justice, and universal standards—create a “strong situation” that restricts individual variability. As evidenced by the non-significant slope in Wave 2, a strong ethical infrastructure effectively overrides personal inclinations. It does not necessarily change the individual’s values, but it neutralizes their negative behavioral expression by raising the cost of deviance and making rule adherence the only acceptable path.

The absence of a significant interaction between Self-Enhancement and Principled Climate on OCB (H6b), along with the null direct effects for SE (H2b), reinforces the distinction between prescriptive and proscriptive morality (Janoff-Bulman et al., 2009). A Principled climate is effective at proscription (inhibiting CWB via rules) but may not provide the necessary cues to stimulate prescription (promoting OCB) among self-enhancing individuals. Individuals high in SE are typically driven by instrumental motives such as impression management or reward (Bolino et al., 2006; Rioux & Penner, 2001). A Principled climate, which emphasizes duty and the “greater good” rather than individual gain, appears insufficient, on its own, to activate the instrumental motives typically associated with self-enhancement. Thus, while the climate successfully stops them from doing bad (CWB), it does not necessarily inspire them to do good (OCB).

Furthermore, Table 4 shows that the effects associated with OCB are generally weaker in both waves compared to CWB. This asymmetry suggests that OCB and CWB are distinct constructs requiring differentiated approaches (Spector & Fox, 2010). The omission of ethical behaviors is less visible and less sanctioned than the commission of unethical acts (Haidt & Baron, 1996). Consequently, organizational climate appears to be a stronger “brake” for bad behavior than a “fuel” for good behavior, particularly when individual values are not naturally aligned with the collective good.

Finally, a key contribution of this work is the stability of these mechanisms over time. The replication of the Activation, Suppression, and Neutralization patterns in Wave 2—often with larger effect sizes—validates the temporal precedence of the model. This confirms that the interplay between values and climate is not a transient state but a stable regulatory feature of the employee-organization relationship. By separating predictors (Wave 1) and outcomes (Wave 2), we minimize common method bias and provide robust evidence that value-climate fit/misfit is a reliable predictor of future ethical conduct.

### 5.1. Theoretical Implications

This study advances the literature on behavioral ethics in three key ways. First, it responds to calls to integrate individual and contextual antecedents by adopting a Dynamic Person–Organization Fit approach. Unlike traditional fit models that conceptualize fit as a static outcome, our findings show that the interaction between personal values and ethical climate activates distinct regulatory mechanisms—Activation, Suppression, and Neutralization—depending on the specific value–climate configuration.

Second, this research provides empirical support for the normative reformulation of the Ethical Climate 2.0 model (Weber & Opoku-Dakwa, 2022). By operationalizing ethical climates as hierarchical moral preferences rather than merely descriptive organizational routines, our results demonstrate that a Principled climate functions as a strong situation capable of neutralizing the behavioral expression of self-enhancement values. This finding reinforces the theoretical claim that ethical climate operates as a normative structure that constrains moral agency, rather than as a passive contextual descriptor.

Third, this study extends Trait Activation Theory into the domain of organizational ethics. Our findings indicate that egoism-oriented climates do not simply discourage ethical behavior; rather, they can activate the moral self-concept of individuals high in Self-Transcendence, prompting resistance to situational pressures. This pattern highlights the resilience of prosocial values and suggests that adverse ethical environments may paradoxically strengthen value-consistent behavior among individuals with strong moral identities.

Taken together, these contributions advance ethical climate theory by demonstrating that climate hierarchy matters for understanding how personal values translate into behavior. Ethical climates function as normative regulators that differentially enable, suppress, or neutralize the behavioral expression of values, thereby offering greater explanatory leverage than purely descriptive climate typologies. In this sense, the present study empirically advances Weber and Opoku-Dakwa’s proposal to reconceptualize ethical climate as a prescriptive organizational force shaping ethical and unethical behavior.

### 5.2. Practical Implications

The internal and external valuation of organizations largely depends on the stability of their reputation and ethical standing. Yet, many institutions face the persistent challenge of unethical behaviors among employees or management, which not only harm organizational functioning but also undermine stakeholder trust. Consequently, both public and private organizations have implemented initiatives aimed at preventing unethical conduct and fostering prosocial behaviors.

The present study contributes to these efforts by showing that ethical behavior can be enhanced when ethical climate dimensions are intentionally aligned with the dominant personal values of employees. Beyond the direct effects of values and perceived ethical climate on conduct, our results highlight the moderating role of person–organization fit, underscoring that congruence between individual values and contextual ethical cues is a key mechanism through which organizations can promote or inhibit ethical functioning.

*Managing Values and Climate Alignment.* Personal values are motivational constructs that shape individuals’ preferences and decisions (Schwartz, 1992). In this framework, organizational citizenship behaviors (OCB) or counterproductive work behaviors (CWB) are expressions of choices that employees perceive as consistent—or inconsistent—with their motivational priorities. Therefore, organizations should deliberately define the types of value orientations they seek to cultivate and maintain. Competitive dynamics can be healthy in moderation, but if they dominate institutional discourse, they risk fostering egoism ethical climates. Managers should thus ask: What values do we seek in our members? Which values should we reinforce internally? Do we know the actual values of our employees?

Following the principle that what is not measured cannot be managed, organizations can assess personal values both at entry and throughout employment. Questionnaires derived from Schwartz’s Value Theory provide reliable tools for this purpose. Although values are relatively stable, they can be activated or deactivated by situational and organizational cues (Verplanken & Holland, 2002). Thus, targeted communications, leadership practices, and workplace design can intentionally reinforce the activation of prosocial values aligned with the organization’s ethical aspirations.

*Selection and Recruitment.* Value-based assessments can be incorporated into the hiring process to identify applicants whose motivational orientations fit the organization’s ethical climate. For example, teams operating in a principled climate may benefit from individuals high in self-transcendence (Arieli et al., 2020).

*Onboarding and Socialization.* Induction programs can explicitly communicate ethical expectations and reinforce desired climate dimensions (e.g., integrity, fairness, collective responsibility). Introducing ethical dilemmas or guided discussions during onboarding helps connect institutional norms with employees’ personal values (Newman et al., 2017).

*Leadership Development.* Ethical leadership sustains the congruence between climate and values. Leaders who articulate moral reasoning and recognize prosocial conduct model value-consistent behavior and reinforce principled climates (Banks et al., 2020).

*Performance Management and Feedback.* Evaluation systems can integrate ethical indicators that reflect the organization’s climate—such as transparency, moral consistency, and supportive behavior—allowing early identification of misalignments between values and expectations.

*Organizational Culture Design.* Ethical climate should be seen as a strategic lever rather than a static descriptor. Through value audits, organizations can assess team-level motivational profiles and adjust climate dimensions accordingly. Reinforcing shared norms of justice and accountability, especially in contexts with diverse value orientations, can improve cohesion and reduce counterproductive behaviors (Weber & Opoku-Dakwa, 2022).

*Contextual and Strategic Relevance.* From a person–organization fit perspective, an egoism climate may weaken the influence of prosocial values on ethical behavior, while a principled climate grounded in justice and collective welfare can buffer the effects of self-enhancement values on unethical conduct. Managing this fit means fostering configurations that

(a) inhibit negative impulses (e.g., principled climate with self-enhancement values),
(b) prevent the suppression of prosocial motivations (e.g., non-egoism climate with self-transcendence values), and
(c) actively strengthen ethical tendencies through situational activation of prosocial values (e.g., principled climate reinforcing self-transcendence).

These insights are particularly relevant in mid-development institutional contexts such as Chile, where ethical guidelines are becoming increasingly formalized, but cultural and motivational diversity still pose challenges (Ralston et al., 2011). Because the study used a longitudinal design—with predictors measured in Wave 1 and behavioral outcomes in Wave 2—its conclusions are temporally robust and applicable to organizations seeking to shape ethical behavior proactively and sustainably over time.

### 5.3. Limitations and Further Research

Our study has several limitations that should be considered when interpreting the findings.

First, although the semi-longitudinal design strengthens the temporal validity of the results, participant attrition in Wave 2 represents a limitation. Attrition analyses indicated that participants who dropped out showed slightly higher self-transcendence, while no significant differences were observed for the remaining key variables. Overall, the follow-up sample can be considered sufficiently comparable; however, the reduced sample size warrants caution when interpreting moderation effects. Future research should seek to replicate these findings using larger and more stable panel samples.

Second, the use of self-report measures entails potential limitations, as motivated biases such as social desirability and consistency seeking may distort responses (e.g., Mõttus et al., 2012; Paulhus & Vazire, 2009). In the present study, these concerns were explicitly addressed a priori through several methodological decisions. A substantial body of literature supports the validity of self-reports in organizational and behavioral research (Spector, 1992). Moreover, previous studies suggest that self-reports may even enhance criterion-related validity, particularly when the constructs of interest involve internal states, intentions, or self-regulatory processes, as is the case with personal values and ethical behavior (Ones et al., 1993; Sassenberg & Ditrich, 2019). Participants were assured full anonymity, and the sample was composed of workers who did not belong to the same organization. This design feature reduces evaluation apprehension and minimizes incentives to present oneself in a socially desirable manner, as responses could not entail reputational, interpersonal, or organizational consequences. Consequently, the likelihood of impression management or strategic responding was substantially reduced. A distraction task was deliberately introduced between the measurement of independent variables (personal values and ethical climate) and dependent variables (OCB and CWB). This procedural separation has been recommended as an effective strategy to limit consistency motives and common method bias, as it disrupts respondents’ ability to infer the study’s hypotheses or maintain cognitive alignment across responses (Schwartz et al., 2017). Finally, the semi-longitudinal design of the study provides an additional safeguard against common method bias. While all predictors were measured at Wave 1, the criterion variables were reassessed independently in Wave 2, three to five months later. This temporal separation not only reduces systematic response tendencies but also allows the cross-sectional findings from Wave 1 to be corroborated over time, strengthening the robustness of the observed relationships.

Third, despite the longitudinal component, the study did not include experimental manipulation of ethical climate or personal values; therefore, causal inferences cannot be drawn. Future research would benefit from longitudinal designs with three or more measurement waves, as well as from experimental or quasi-experimental approaches in laboratory or field settings, to more precisely disentangle causal mechanisms linking values, ethical climate, and behavior (Treviño et al., 2014).

Fourth, for theoretical and practical reasons, the present study focused on two higher-order value dimensions of Schwartz’s model and on situations of value–climate incongruence. While this strategy allowed for a clearer test of regulatory mechanisms, future studies with larger samples could examine congruent configurations and incorporate additional value dimensions, such as Openness to Change and Conservation.

Fifth, future research should extend this framework to other ethically ambiguous or mixed-motive behaviors, such as unethical pro-organizational behavior (Mishra et al., 2022), and further explore the conditions under which Organizational Citizenship Behavior may transition into Counterproductive Work Behavior (Klotz & Bolino, 2013).

Sixth, although the hypotheses were theoretically specified in advance, some of the more fine-grained directional claims concerning activation, suppression, neutralization, and alignment mechanisms would ideally benefit from confirmation through pre-registered designs. Given the longitudinal component and the relatively modest sample size at Wave 2, replication with larger samples and, where feasible, pre-registered hypotheses and analytic strategies would further strengthen the evidentiary basis of these interaction effects and their directional interpretation.

Finally, the cultural context of the study should be acknowledged. Data were collected exclusively in Chile, a country characterized by specific cultural value orientations that may shape how ethical climates are perceived and enacted (Ralston et al., 2011). Consequently, the generalizability of the findings to other cultural settings remains an open question. Future cross-cultural research should examine whether the activation and neutralization mechanisms identified in this study operate similarly across more individualistic or collectivist societies.

## 6. Conclusions

In summary, the results obtained in this work contribute significantly to the challenge of managing (un)ethical behavior in organizations, providing plausible guidelines to address this issue from a person-in-context approach. Although some other research supports—separately—the role of personal values and ethical climate as antecedents of (un)ethical behavior (Arciniega et al., 2019; Bolino et al., 2006; Pulfrey & Butera, 2013; Ring et al., 2022; Rioux & Penner, 2001), this study states the potential of complementing these models to achieve a more precise and efficient approach to this kind of behavior, which is the basis of good organizational performance. Likewise, this work contributes to the recent and growing interest in analyzing the moderating role of organizational factors, such as the ethical climate, in the relationship between personal variables and (un)ethical behavior (Treviño et al., 2014; Newman et al., 2017). Consequently, Schwartz’s theory of personal values and Victor and Cullen’s perceived ethical climate model should be considered as facets of the person–organization space, whose characteristics and complementary power must be further studied and applied to better understand and manage organizational (un)ethical behavior.

## Figures and Tables

**Table 1:** Demographic information of the participants.

Parameters	Wave 1	Wave 2
Total sample, *n*	212	84
Gender, *n* (%)		
Female	98 (46.2)	39 (46.4)
Male	112 (52.8)	45 (53.6)
Other	2 (1.0)	-
Mean age in years, *M* (*SD*)	37.29 (11.86)	40.63 (13.42)
Occupational status, *n* (%)		
Full-time job	161 (75.9)	61 (72.6)
Part-time job	38 (17.9)	16 (19)
Independent worker	8 (3.8)	1 (1.2)
Apprenticeship contract	3 (1.4)	2 (2.4)
Informal employment	2 (1.0)	4 (4.8)
Completed educational level, *n* (%)		
Graduate studies	49 (23.1)	27 (32.2)
Undergraduate studies	87 (41)	38 (45.2)
Technical/professional studies	47 (22.2)	10 (11.9)
High school	27 (12.7)	8 (9.5)
Elementary school	2 (1.0)	1 (1.2)
Organization size, *n* (%)		
1 to 9 workers	28 (13.2)	14 (16.7)
10 to 49 workers	20 (9.4)	6 (7.2)
50 to 199 workers	40 (18.9)	16 (19)
200 or more workers	124 (58.5)	48 (57.2)
Occupational level, *n* (%)		
Manager/Director	20 (9.4)	10 (11.9)
Chief/Supervisor	52 (24.5)	22 (26.2)
Administrative	77 (36.3)	30 (35.7)
Laborer	63 (29.7)	22 (26.2)
Modality of work, *n* (%)		
Face-to-face	134 (63.2)	45 (53.6)
Hybrid	63 (29.7)	8 (9.5)
Teleworking	15 (7.1)	31 (36.9)
Seniority in the organization, *M* (*SD*)		9.35 (9.7)
Region of residence in Chile (most represented), *n* (%)		
Metropolitan area of Santiago	89 (42)	31 (36.9)
La Araucanía	54 (25.5)	33 (39.3)
Bio-Bio	34 (11.3)	12 (14.3)
Other	35 (16.5)	8 (9.5)

**Table 2:** Mean, standards deviation, Cronbach’s alpha, McDonald’s omega, square root of average variance extracted (AVE) and variable correlations (*n* = 212).

Variable	*Mean*	*SD*	*α*	*ω*	1	2	3	4	5	6
1. ST	6.426	0.510	0.648	0.647	(0.530)	0.007	−0.126	0.242 **	0.392 **	−0.282 **
2. SE	4.706	1.050	0.817	0.826		(0.733)	0.166 *	0.106	0.079	0.307 **
3. E-C	4.301	1.313	0.803	0.809			(0.684)	−0.364 **	−0.092	0.180 **
4. P-C	4.654	0.892	0.784	0.788				(0.658)	0.297 **	−0.120 †
5. OCB	3.889	0.604	0.778	0.771					(0.576)	−0.357 **
6. CWB	1.382	0.411	0.756	0.765						(0.522)

*Note*. † *p* < 0.10; * *p* < 0.05, ** *p* < 0.01; (___) = Square root of AVE; ST = Self-Transcendence; SE = Self-Enhancement; E-C = Egoism climate; P-C = Principled climate; OCB = Organizational Citizenship Behavior; CWB: Counterproductive Work Behavior.

**Table 3:** CFA fit indices for the single-factor and the theoretical six-factor model.

Model 1	*χ* ^2^	*df*	*p*	*χ*^2^/*df* Ratio	SRMR	RMSEA [90% CI]	CFI	TLI
Single-factor model	2150.599	665	<0.001	3.23	0.117	0.103 [0.098–0.108]	0.628	0.607
Initial six-factor model	862.805	725	<0.001	1.19	0.071	0.030 [0.021–0.038]	0.966	0.964
Final six-factor mode (*)	599.437	579	<0.001	1.03	0.065	0.013 [0.000–0.026]	0.995	0.994

*Note***.** *N* = 212; *χ*^2^ = chi square; *df* = degree of freedom; SRMR = Standardized Root Mean Residual; RMSEA = Root Mean Square Error of Approximation; CFI = Comparative Fit Index; TLI = Tucker–Lewis Index. (*): The final model discarded four items with low factor loadings.

**Table 4:** Hypotheses testing results: standardized regression coefficients (*β*), *p*-values, variance inflation factors (VIF), and *R*^2^ for interaction models (Waves 1 and 2).

	Wave 1		VIF	*R* ^2^	Wave 2		VIF	*R* ^2^
	Std. Coeff.	*p*-Value			Std. Coeff.	*p*-Value		
H1a: Direct effect ST-CWB	−0.261	<0.001 **	1.18		−0.289	0.007 **	1.35	
H3a: Direct effect Egoism climate–CWB	0.140	0.038 *	1.07		0.252	0.016 *	1.04	
H3b: Moderation of Egoism climate on ST-CWB	−0.255	<0.001 **	1.18	0.160	−0.513	<0.001 **	1.29	0.411
H2a: Direct effect ST-OCB	0.385	<0.001 **	1.18		0.211	0.070 †	1.35	
H4a: Direct effect Egoism climate–OCB	−0.030	0.641	1.07		0.040	0.722	1.04	
H4b: Moderation of Egoism climate on ST-OCB	−0.135	0.050 †	1.18	0.178	−0.259	0.036 *	1.29	0.111
H1b: Direct effect SE-CWB	0.318	<0.001 **	1.05		0.325	0.003 **	1.15	
H5a: Direct effect Principled climate–CWB	−0.151	0.023 *	1.02		−0.214	0.040 *	1.09	
H5b: Moderation of Principled climate on SE-CWB	−0.133	0.043 *	1.02	0.136	−0.261	0.016 *	1.15	0.240
H2b: Direct effect SE-OCB	0.062	0.355	1.05		0.178	0.128	1.15	
H6a: Direct effect Principled climate–OCB	0.285	<0.001 **	1.02		0.050	0.651	1.09	
H6b: Moderation of Principled climate on SE-OCB	0.085	0.203	1.02	0.106	−0.026	0.829	1.15	0.047

*Note.* Wave 1: *n* = 212; Wave 2: *n* = 84. VIF = Variance Inflation Factor. *R*^2^ is only reported for models including interaction terms. † *p* < 0.10; * *p* < 0.05; ** *p* < 0.01.

**Table 5:** Simple slope analysis and interaction effect sizes comparing Wave 1 and Wave 2.

	Wave 1		Wave 2	
Moderator Condition	Slope (Std. Coeff.)	*p*-Value	Slope (Std. Coeff.)	*p*-Value
Moderation by value activation (Self-Transcendence × Egoism → CWB)				
Low Egoism (−1 SD)	0.07	0.540	0.42	0.012 *
High Egoism (+1 SD)	−0.40	<0.001 **	−0.51	<0.001 **
Interaction Term	Δ*R*^2^ = 0.055	<0.001 **	Δ*R*^2^ = 0.20	<0.001 **
Moderation by value suppression (Self-Transcendence × Egoism → OCB)				
Low Egoism (−1 SD)	0.56	<0.001 **	0.57	0.006 **
High Egoism (+1 SD)	0.31	<0.001 **	0.10	0.417
Interaction Term	Δ*R*^2^ = 0.016	0.050 †	Δ*R*^2^ = 0.052	0.036 *
Moderation by value neutralization (Self-Enhancement × Principles → CWB)				
Low Principled Climate (−1 SD)	0.42	<0.001 **	0.50	<0.001
High Principled Climate (+1 SD)	0.18	0.060 †	0.04	0.793
Interaction Term	Δ*R*^2^ = 0.017	0.043 *	Δ*R*^2^ = 0.059	0.016 *

*Note.* Wave 1: *n* = 212; Wave 2: *n* = 84. Std. Coeff. = Standardized Coefficient; CWB = Counterproductive Work Behavior; OCB = Organizational Citizenship Behavior; SD = Standard Deviation. Δ*R*^2^ represents the unique variance explained by the interaction term. † *p* < 0.10; * *p* < 0.05; ** *p* < 0.01.

## Data Availability

The original contributions presented in this study are included in the article. Further inquiries can be directed to the corresponding authors.

## Appendix A. Questionnaires

**Table A1:** Items comprising the Personal Values Questionnaire (PVQ-21). Based on Schwartz et al. (2001) and validated Spanish version by Beramendi and Zubieta (2017). Items are grouped by higher-order dimension.

English Version	Spanish Version	Dimension
He/She thinks it is important that every person in the world be treated equally. He/She believes everyone should have equal opportunities in life.	Piensa que es importante que a todas las personas del mundo se las trate con igualdad. Cree que todos deberían tener las mismas oportunidades en la vida.	Self-Transcendence
It is important to him/her to listen to people who are different from him/her. Even when he/she disagrees with them, he/she still wants to understand them.	Es importante para él/ella escuchar a las personas que son diferentes. Aún cuando está en desacuerdo con ellas, quiere entenderlas.	Self-Transcendence
It’s very important to him/her to help the people around him/her. He/She wants to care for their well-being.	Es muy importante para él/ella ayudar a la gente que lo/a rodea. Se preocupa por su bienestar.	Self-Transcendence
It is important to him/her to be loyal to his/her friends. He/She wants to devote himself/herself to people close to him/her.	Es importante para él/ella ser leal. Quiere dedicarse a las personas cercanas a él/ella.	Self-Transcendence
He/She strongly believes that people should care for nature. Looking after the environment is important to him/her.	Cree firmemente que las personas deben cuidar la naturaleza. Proteger el medio ambiente es importante para él/ella.	Self-Transcendence
It is important to him/her to be rich. He/She wants to have a lot of money and expensive things.	Para él/ella es importante ser rico/a. Quiere tener mucho dinero y cosas caras.	Self-Enhancement
It’s very important to him/her to show his/her abilities. He/She wants people to admire what he/she does.	Para él/ella es muy importante mostrar sus habilidades. Quiere que la gente admire lo que hace.	Self-Enhancement
Being very successful is important to him/her. He/She hopes people will recognize his/her achievements.	Para él/ella es importante ser una persona muy exitosa. Espera que la gente reconozca sus logros.	Self-Enhancement
It is important to him/her to get respect from others. He/She wants people to do what he/she says.	Para él/ella es importante ser respetado por la gente. Desea que las personas hagan lo que les dice.	Self-Enhancement
Thinking up new ideas and being creative is important to him/her. He/She likes to do things in his/her own original way.	Tener ideas nuevas y ser creativa/o es importante para él/ella. Le gusta hacer las cosas a su manera, de forma original.	Openness
He/She likes surprises and is always looking for new things to do. He/She thinks it is important to do many different things in life.	Le gustan las sorpresas y siempre está buscando cosas nuevas para hacer. Piensa que es importante hacer muchas cosas diferentes en la vida.	Openness
Having a good time is important to him/her. He/She likes to “spoil” himself/herself.	Pasarlo bien es importante para él/ella. Le gusta darse los gustos.	Openness
It is important to him/her to make his/her own decisions about what he/she does. He/She likes to be free and not depend on others.	Para él/ella es importante tomar sus propias decisiones sobre lo que hace. Le gusta ser libre y no depender de otros.	Openness
He/She looks for adventures and likes to take risks. He/She wants to have an exciting life.	Anda siempre en busca de aventuras y le gusta arriesgarse. Tener una vida llena de emociones es importante para él/ella.	Openness
He/She seeks every chance he/she can to have fun. It is important to him/her to do things that give him/her pleasure.	Busca cualquier oportunidad para divertirse. Es importante para él/ella hacer cosas que le den placer.	Openness
It is important to him/her to live in secure surroundings. He/She avoids anything that might endanger his/her safety.	Es importante para él/ella vivir en un entorno seguro. Evita todo lo que pueda poner en peligro su seguridad.	Conservation
He/She believes that people should do what they’re told. He/She thinks people should follow rules at all times, even when no-one is watching.	Cree que las personas deben hacer lo que se les dice. Opina que la gente debe seguir las reglas en todo momento, incluso cuando nadie la está mirando.	Conservation
It is important to him/her to be humble and modest. He/She tries not to draw attention to himself/herself.	Es importante para él/ella ser humilde y modesto/a. Trata de no llamar la atención.	Conservation
It is very important to him/her that the government ensures his/her safety against all threats. He/She wants the state to be strong so it can defend its citizens.	Es importante para él/ella que el gobierno le proteja contra todos los peligros. Quiere que el Estado sea fuerte para que defienda a sus ciudadanos.	Conservation
It is important to him/her always to behave properly. He/She wants to avoid doing anything people would say is wrong.	Es importante para él/ella comportarse siempre correctamente. Procura evitar hacer cualquier cosa que otros juzguen incorrecto.	Conservation
Tradition is important to him/her. He/She tries to follow the customs handed down by his/her religion or his/her family.	La tradición es importante para él/ella. Trata de seguir las costumbres que le han transmitido su religión o su familia.	Conservation

**Table A2:** Items comprising the Principled and Egoism organizational ethical climates. Based on Weber and Opoku-Dakwa (2022).

English Version	Spanish Version	Dimension
When making decisions, people in this organization are encouraged to weigh justice, fairness, human rights, and the effects of their actions on the greater good.	Al tomar decisiones, se alienta a las personas de esta organización a sopesar la justicia, la equidad, los derechos humanos y los efectos de sus acciones en el bien común.	Principled
In this organization, people are expected to be fair and act in the interest of society and humanity.	En esta organización, se espera que las personas sean justas y actúen en interés de la sociedad y la humanidad.	Principled
In this organization, people are expected always to do what is right for society.	En esta organización, se espera que las personas hagan siempre lo que es correcto para la sociedad.	Principled
A respect for the rights of all is a major consideration in this organization.	El respeto por los derechos de todos es una consideración importante en esta organización.	Principled
The long-term effects of decisions and actions are a major consideration in this organization.	Los efectos a largo plazo de las decisiones y acciones son una consideración importante en esta organización.	Principled
(*) In this organization, we take responsibility for the impacts of our actions regardless of who is affected.	(*) En esta organización, asumimos la responsabilidad por el impacto de nuestras acciones sin importar quién se vea afectada/o.	Principled
In this organization, people protect their own interests.	En esta organización, las personas protegen sus propios intereses.	Egoism
People are mostly out for themselves in this organization.	En esta organización, la gente se preocupa principalmente por sí misma.	Egoism
In this organization, each person is responsible for their own survival.	En esta organización, cada persona es responsable de su propia supervivencia.	Egoism
People are expected to protect their own interests in this organization.	Se espera que las personas protejan sus propios intereses en esta organización.	Egoism
Getting ahead is the major consideration in this organization. People protect their own interests.	Avanzar es la consideración principal en esta organización. Las personas protegen sus propios intereses.	Egoism
(*) In this organization, your own safety and personal gain are major considerations.	(*) En esta organización, la seguridad y el beneficio de una/o misma/o son de la mayor importancia.	Egoism

Note. (*) Discarded from the analysis because it showed low factor loadings.

**Table A3:** Items comprising the CWB and OCB measures. Based on Kelloway et al. (2002).

English Version	Spanish Version	Dimension
(*) Exaggerated about your hours worked.	(*) Informó una mayor cantidad de horas trabajadas que las realmente realizadas.	CWB
Started negative rumors about your company.	Inició rumores negativos acerca de su organización.	CWB
Gossiped about your coworkers.	Contó chismes acerca de sus compañeras/ros de trabajo.	CWB
Covered up your mistakes.	Encubrió sus propios errores.	CWB
Competed with your coworkers in an unproductive way.	Compitió con sus compañeras/os de trabajo perjudicando la productividad.	CWB
Gossiped about your supervisor.	Contó chismes acerca de su supervisora/or.	CWB
Stayed out of sight to avoid work.	Se mantuvo no localizable para evitar el trabajo.	CWB
Used organizational equipment or materials for personal use.	Utilizó equipos o materiales de la organización para uso personal.	CWB
Blamed your coworkers for your mistakes.	Culpó a sus compañeras/os de trabajo por los errores que usted cometió.	CWB
Intentionally worked slow.	Trabajó lento intencionalmente.	CWB
Helping other employees with their work when they have been absent.	Ayudar a otras/os empleadas/os con su trabajo cuando han estado ausentes.	OCB
Volunteering to do things not formally required by the job.	Ofrecerse voluntariamente a realizar actividades que no son formalmente requeridas por el puesto de trabajo.	OCB
Taking the initiative to orient new employees to the department even though it is not part of my job description.	Tomar la iniciativa de orientar a las/os nuevas/os empleadas/os del departamento, aunque no forme parte de mis funciones.	OCB
Helping others when their work load increases (assisting others until they get over the hurdles).	Ayudar a otras/os cuando aumenta su carga de trabajo [hasta que resuelvan el problema].	OCB
Assisting supervisor with his/her duties.	Apoyar a la/al supervisora/or en sus funciones.	OCB
Making innovative suggestions to improve the overall quality of the department.	Hacer sugerencias innovadoras para mejorar la calidad general del departamento.	OCB
(*) Punctuality in arriving at work on time in the morning, and after lunch and breaks.	(*) Puntualidad en el horario de inicio de la jornada de trabajo, y al regreso de la colación y de los descansos.	OCB
Exhibiting attendance at work beyond the norm, for example I take less days off than most individuals or less than allowed.	Dedicarle más tiempo al trabajo que el formalmente exigido, por ejemplo, tomando menos tiempo libre que la mayoría, o de lo que está permitido.	OCB
Giving advance notice if unable to come to work.	Dar aviso con anticipación si debe ausentarse del trabajo [presencial o remoto].	OCB

Note. (*) Discarded from the analysis because it showed low factor loadings.
